# Therapeutic Potential of *Lespedeza bicolor* to Prevent Methylglyoxal-Induced Glucotoxicity in Familiar Diabetic Nephropathy

**DOI:** 10.3390/jcm8081138

**Published:** 2019-07-31

**Authors:** Moon Ho Do, Jae Hyuk Lee, Kyohee Cho, Min Cheol Kang, Lalita Subedi, Amna Parveen, Sun Yeou Kim

**Affiliations:** 1 College of Pharmacy, Gachon University, 191, Hambakmoero, Yeonsu-gu, Incheon 21936, Korea; 2 Division of Functional Food Research, Korea Food Research Institute, 245 Nongsaengmyeong-ro, Iseo-myeon, Wanju-gun, Jeollabuk-do 55365, Korea; 3 Gachon Institute of Pharmaceutical Science, Gachon University, 191, Hambakmoero, Yeonsu-gu, Incheon 21936, Korea

**Keywords:** advanced glycation end-products, diabetic nephropathy, hyperglycemia, methylglyoxal, *Lespedeza bicolor*

## Abstract

*Lespedeza bicolor* (LB) is often used in traditional medicine to remove toxins, replenish energy stores, and regulate various symptoms of diabetes. This study aimed to explore the use of LB as a therapeutic to prevent diabetic nephropathy in methylglyoxal (MGO)-treated models in vitro and in vivo. Western blotting, immunostaining, and biochemical assays were used to obtain several experimental readouts in renal epithelial cells (LLC-PK1) and BALB/c mice. These include: production of reactive oxygen species (ROS), formation of advanced glycation end-products (AGEs), expression of receptor for advanced glycation end-products (RAGE), apoptotic cell death, glucose levels, fatty acid and triglyceride levels, expression of pro-inflammatory cytokines IL-1β and TNF-α, glyoxalase 1 (Glo1), and nuclear factor erythroid 2-related factor 2 (Nrf2). Pretreatment with LB significantly reduced MGO-induced cellular apoptosis, intracellular production of ROS, and formation of AGEs to ameliorate renal dysfunction in vitro and in vivo. Interestingly, administering LB in MGO-treated cells and mice upregulated the expression of Nrf2 and Glo1, and downregulated the expression of IL-1β and TNF-α. Moreover, LB reduced MGO-induced AGE accumulation and RAGE expression in the kidneys, which subsequently reduced AGE-RAGE interactions. Overall, LB ameliorates renal cell apoptosis and corrects renal dysfunction in MGO-treated mice. These findings extend our understanding of the pathogenic mechanism of MGO-induced nephrotoxicity and regulation of the AGE/RAGE axis by *Lespedeza bicolor*.

## 1. Introduction

The global prevalence of diabetes mellitus (DM) is projected to increase from 6.4% in 2018 to 7.7% in 2030 [1]. Clinical data demonstrates that nearly one-third of patients with DM suffer from diabetic nephropathy (DN), and there are currently no tests available for early diagnosis. As the disease continues to progress, the kidneys become incapable of clearing drugs and toxins from the body, which can be harmful to patient health, leading to severe illness or death. Numerous causative factors are known to initiate signaling pathways involved in the DN pathogenesis [2]. 

The levels of methylglyoxal (MGO), which is an important precursor in the formation of advanced glycation end-products (AGEs), are increased in hyperglycemic patients [3]. Various DN clinical studies indicate that the increase in MGO levels induces dicarbonyl accumulation. This accumulation results in dicarbonyl stress, which leads to the formation of AGEs and is ultimately responsible for the pathogenesis of atherosclerosis, retinopathy, and renal dysfunction. Activated glyoxalase 1 (Glo1) detoxifies MGO, which ultimately inhibits the formation of AGEs [4,5]. 

Previous studies indicate that MGO can induce cellular apoptosis by increasing production of reactive oxygen species (ROS), which has deleterious effects on the kidneys. Decreased Glo1 activity, under conditions of high glucose concentration, results in MGO accumulation and subsequent formation of AGEs that activate the receptor of AGEs (RAGE), suggesting a possible role of Glo1 in the pathogenesis of DN [6]. In the kidneys, Glo1 activity is regulated by nuclear factor erythroid 2-related factor 2 (Nrf2), which is associated with the altered renal structure and renal dysfunction. Furthermore, Nrf2 is an important constituent of antioxidant responsive element (ARE)-mediated transcriptional induction and is, therefore, a critical player in cellular responses to oxidative stress. Thus, activation of Nrf2 downregulates MGO accumulation and upregulates Glo1 expression, which may be beneficial for treating DN [7,8]. These observations reveal a pathogenic role of MGO in DN, and reveal a potential therapeutic route in delaying DN onset as its production can be targeted with multiple signaling molecules.

At present, no drugs are available for effectively preventing and treating DN. Medicinal plants could be a safer alternative for the treatment of DN, due to the presence of various active compounds with multiple pharmacological effects [9]. Previous studies reveal that natural products or naturally derived compounds—such as *Eucommia ulmoides*, diphlorethohydroxycarmalol (DPHC), and catechin—exhibit antidiabetic activity targeting MGO-associated DN [10,11,12]. 

*Lespedeza bicolor* (LB) (Leguminosae) plants used in traditional medicine for the treatment of azotemia, nephritis, diuresis, diabetes, energy depletion, and inflammation [13,14]. Lee et al. reported previously that LB is effective in treating ailments such as a cough, fever, dieresis, azotemia, acute and chronic nephritis, and estrogenic activity [15,16,17]. H.S. Woo et al. reported that root bark of LB contained significant bacterial neuraminidase inhibitory activity [18]. It has recently been demonstrated that *Lespedeza* species can protect pancreatic β-cells from cytokine-induced damage in streptozotocin-induced diabetic mice [19,20], inhibit the formation of AGEs [21], and ameliorate endothelial dysfunction arising from MGO-induced glucotoxicity in diabetic retinopathy mode [13]. Chemical composition from LB extract includes stigmasterols, organic acids, terpenes, alkaloids, and flavonoids that have been screened for anticancer, anti-radiation, antioxidant, reducing blood sugar, and anti-inflammatory activity. Our recent studies indicate that main compounds from LB extract such as genistein, quercetin, and naringin were considered to inhibit the formation of AGEs [13]. However, the mechanism behind the potential effect of LB on MGO-induced diabetic nephropathy has not been studied. In this study, we examined how the recovery of MGO-induced metabolic dysfunction and glucotoxicity cane be done using in vitro and in vivo models with *Lespedeza bicolor* pretreatment and its related underlying mechanism targeting Glo1 and Nrf2 upregulation.

## 2. Material and Methods

### 2.1. Chemicals and Reagents

O-phenylenediamine (o-PD), perchloric acid, 2′,7′-dichlorofluorescein diacetate (DCFH-DA), bovine serum albumin (BSA), MGO, 3-(4,5-dimethylthiazol-2-yl)-2,5-diphenyltetrazolium bromide (MTT), dimethyl sulfoxide (DMSO), catechin, rutin, luteolin, naringenin, and genistein were purchased from Sigma (St. Louis, MO, USA). Daidzein was obtained from LC Laboratories (Wobum, MA, USA). The LLC-PK1 cell line and fetal bovine serum (FBS) were purchased from American Type Culture Collection (Rockville, MD, USA). Dulbecco’s modified Eagle’s medium (DMEM) was purchased from HyClone (Logan, Utah, USA). Antibodies against Nrf2, Glo1, RAGE, and α-tubulin were purchased from Santa Cruz Biotechnology (Dallas, TX, USA). The antibody against AGE was procured from Abcam (Cambridge, MA, USA), 2-methylquinoxaline (2-MQ), and 5-methylquinoxaline (5-MQ) was purchased from Tokyo Chemical Industries (Tokyo, Japan), while 3-(4,5-dimethylthiazol-2-yl)-2,5-diphenyltetrazolium bromide (MTT) and Pro-Prep^TM^ were purchased from iNtRON Biotechnology (Seongnam, Korea).

### 2.2. Preparation of LB Extract

*Lespedeza bicolor* (LB) plants were purchased from Jayeonchunsa Co., Damyang, Republic of Korea. The LB stalks were identified by Dr. Kim Sun Yeou, and the voucher specimen (number KSY-HP-008-033) was deposited at the College of Pharmacy at Gachon University. The powdered plant material was extracted with 70% ethanol, filtered, dried with a rotary evaporator, and kept at 4 °C until further analysis. The study detailing the preparation of LB extract, as well as a description of phytochemicals through LC-MS analysis, has been detailed in our recently published paper [13]. 

### 2.3. Quantitative Study of the Phytochemicals in LB

The quantitative study of the phytochemicals in the was investigated by HPLC. The HPLC systems (Waters Corp., Milford, MA, USA) comprised of an autosampler, separation modules (e2695) with a photodiode array detector. The separation was performed using a column C18 (250, 4.6 mm × 5 µm). The chromatographic conditions are the following: a gradient mobile phase consists of ACN (A) and 0.1% phosphoric acid (B): 7→90% A was run for 60 min at a temperature of 30 °C at a flow rate of 1 mL/min. Injection volume was 10 and chromatography was detected at a wavelength of 283 nm. A mixture of standard compounds named rutin, catechin, genistein, daidzein, naringenin, and luteolin were used at a concentration of 1mg/mL to identify peaks. 

### 2.4. Cell Culture

Kidney epithelial LLC-PK1 cells were maintained under standard cell culture conditions, at 37 °C in a humidified incubator with 5% CO_2_. The cells were cultured in DMEM supplemented with 10% FBS and penicillin-streptomycin solution (100 U/mL).

### 2.5. MTT Assay

In order to determine cell viability with the MTT assay, LLC-PK1 cells were seeded in a 96-well plate at a density of 1.0 × 10^4^ cells/well and incubated at 37 °C. After one day of incubation, the cells were pretreated with LB for 1 h while the cells in the control group were left untreated. Following this for 1 h, all the cells were incubated with MGO for 24 h. After incubation, MTT solution (0.1 mg/mL) was added to each well, and the cells were incubated for 2 h. The medium was then gently removed and 100 μL of DMSO was added to the wells. A microplate reader (Molecular Devices, CA, USA) was used to determine the absorbance at a wavelength of 570 nm. Aminoguanidine (AG) at a dose of 1 mM was used as a positive control. 

### 2.6. Cell Apoptosis Assay

The Annexin V Kit was used to determine the effect of LB on early and late apoptosis of LLC-PK1 cells after MGO induction. The cells were seeded in a 6-well plate at a density of 5.0 × 10^5^ cells/well and incubated for 24 h at 37 °C. Following incubation, the cells were pretreated with LB and then after 1 h MGO was added and incubated for a second time for 24 h. The cells were subsequently stained with Annexin V-FITC. A flow cytometer was used to analyze apoptosis in LLC-PK1 cells. Aminoguanidine (AG) at a dose of 1 mM was used as a positive control.

### 2.7. Measurement of Intracellular Oxidative Stress 

To determine the effect of LB on oxidative stress induced by elevated levels of ROS, LLC-PK1 cells were seeded in a 6-well plate and incubated at 37 °C for 24 h. The cells were subsequently pretreated with LB and incubated for 1 h, then treated with MGO for 24 h. The cells were then washed with PBS and incubated in 10 µM 2′,7′-dichlorofluorescein diacetate (DCF-DA) at 37 °C for 20 min. The cells were washed again with PBS, and fluorescence intensity was measured with a flow cytometer. Aminoguanidine (AG) at a dose of 1 mM was used as a positive control.

### 2.8. Western Blotting 

Western blot analysis was performed to determine changes in protein levels in LLC-PK1 cells. The cells that had been pretreated with LB were incubated with MGO for 1, 6, and 24 h. Pro-Prep^TM^, which contains a phosphatase inhibitor, was used for extracting the total protein in the cells. Protein concentration was determined with a Bradford assay. An equal amount of protein was loaded into the wells on a sodium dodecyl sulfate (SDS)-polyacrylamide gel, and electrophoresis was carried out for 80 min. The gel was then transferred to a nitrocellulose membrane and blocked using 5% skim milk. After 1 h, the membrane was incubated with primary antibodies on a rocking tray in the refrigerator. In order to visualize the immunoreactive proteins, peroxidase-labeled secondary antibodies were used, and the immunoblots were visualized using a chemiluminescent reagent. The relative intensities of the protein bands were quantified with the ChemiDoc XRS + imaging system (Bio-Rad, Hercules, CA, USA). 

### 2.9. Animal Experiments

Four-week-old male BALB/c mice were procured from Orient Bio Inc. (Seongnam, Korea) and were housed under controlled environmental conditions, where the temperature was maintained at 23 °C, and the relative humidity was 65% under a 12 h light/12 h dark cycle. The mice were allowed ad libitum access to food and water. The animals were acclimated for one week prior to testing. All the procedures and experiments were approved by the animal ethics committee at the Lee Gil Ya Cancer and Diabetes Institute of Gachon University, Korea (LCDI-2016-0071). The animals were divided into the following four groups, each containing six mice: (1) normal, (2) the MGO-treated group (300 mg/kg), (3) the MGO+LB-treated group (10 mg/kg), and (4) the MGO+LB-treated group (100 mg/kg). MGO and LB were orally administered daily for 28 days. 

### 2.10. Oral Glucose Tolerance Test (OGTT)

OGTT was performed after 27 days of treatment. The animals were fasted overnight (24 h), following which glucose was administered at a dose of 1 g/kg of the bodyweight (BW). The blood glucose level was subsequently noted at intervals of 0, 30, 60, 90, and 120 min from the administration of glucose with a glucometer. 

### 2.11. Analysis of Plasma Lipids

Blood was collected through the retro-orbital venous plexus into EDTA-coated tubes and centrifuged for 15 min at 3000 rpm. The levels of fatty acids and triglycerides were determined with ELISA kits (Abcam, Cambridge, MA, USA). The level of cytokines was determined according to the manufacturer instructions, and the absorbance was determined with a microplate reader (Molecular Devices). 

### 2.12. Determination of MGO Levels in the Kidneys

High-performance liquid chromatography (HPLC, Waters system, Waters Corp., Milford, MA, USA) was performed in order to determine the levels of MGO in the kidneys according to the protocol by Dhar et al. [22]. The amount of unreacted MGO in the samples was estimated by the ratio of the peak area of 2-methylquinoxaline (2-MQ) to that of 5-methylquinoxaline (5-MQ). It is known that o-PD reacts with MGO to form 2-MQ. 2-MQ and 5-MQ were considered as the external and internal standards, respectively. The kidney tissues were homogenized and incubated for 24 h with 10 mM O-phenylenediamine (o-PD) and 0.45 N perchloric acid. The samples were then centrifuged for 10 min at 12,000 rpm and filtered through a 0.45-µm membrane filter prior to being injected into the HPLC system. The HPLC system had a separating module (e2695) connected to a photodiode array detector (2998). The samples were analyzed using an INNO column (150 × 460 mm, 5 µm) with 20% acetonitrile as the mobile phase at a detection wavelength of 315 nm. The flow rate of the mobile phase was 1 mL/min. 

### 2.13. Evaluation of the Levels of AGEs, IL-1β, and TNF-α in the Kidneys

The levels of AGEs, IL-1β, and TNF-α were evaluated using ELISA kits purchased from Cell Biolabs, Inc. (Beverly, MA, USA) and R&D Systems (Minneapolis, MN, USA). The protein levels were normalized and determined according to the methods of manufacturer instructions. 

### 2.14. Histology and Immunohistochemistry Studies

Antigens were retrieved to determine the accumulation of AGEs and RAGE expression. In order to reduce peroxidase activity, 4-µm-thick sections of kidney tissues were incubated for 15 min with 3% H_2_O_2_ in PBS, and then incubated with primary antibodies for 12 h at 4 °C. The tissue sections were then washed with PBS and incubated with secondary antibodies for 20 min at room temperature. The sections were washed again with PBS and incubated in Vectastain ABC reagent for 30 min. The immunoreactions were visualized using 3,3′-diaminobenzidine. The sections were counterstained with hematoxylin for 3 min. 

### 2.15. Statistical Analysis

The data are represented as the mean ± standard error of the mean (SEM) and analyzed by one-way analysis of variance (ANOVA), followed by Bonferroni’s test. The level of significance was set at *p* < 0.05 unless otherwise indicated. All experiments were performed in triplicate. 

## 3. Results

### 3.1. LB Pretreatment Attenuated MGO-Induced Cytotoxicity, Apoptosis, and Oxidative Stress in LLC-PK1 Cells

**Figure 1:**
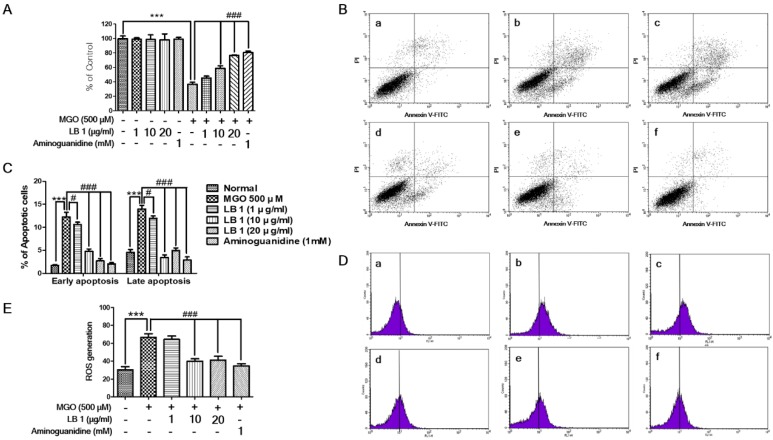
The effect of LB on reduced cell viability and increased apoptosis and oxidative stress induced by MGO in LLC-PK1 cells. (**A**) Viability of LB and MGO-treated LLC-PK1 cells was analyzed with MTT assay. (**B**) Effect of LB on MGO-induced apoptosis of LLC-PK1 cells. Flow cytometry analysis in MGO-stimulated LLC-PK1 cells performed with annexin V-FITC and PI staining: **a**, control; **b**, cells treated with 500 μM MGO; **c**, cells treated with MGO + LB (1 μg/mL); **d**, cells treated with MGO + LB (10 μg/mL); **e**, cells treated with MGO + LB (20 μg/mL); and **f**, cells treated with MGO + aminoguanidine (1 mM) as the positive control. (**C**) Effect of LB on early and late apoptosis was analyzed by flow cytometry. (**D**) The results of flow cytometry performed with DCF-DA staining of MGO-stimulated LLC-PK1 cells: **a**, control; **b**, cells treated with 500 μM MGO; **c**, cells treated with MGO + LB (1 μg/mL); **d**, cells treated with MGO + LB (10 μg/mL); **e**, cells treated with MGO + LB (20 μg/mL); and **f**, cells treated with MGO + aminoguanidine as the positive control. (**E**) Effect of LB on the generation of ROS was analyzed by flow cytometry. The values are represented as the mean ± SEM (##*p* < 0.01 and ### *p* < 0.001 vs. MGO treatment, and ** *p* < 0.01 vs. the control).

Cell cytotoxicity, apoptotic cell death, and production ROS of LLC-PK1 cells were significantly induced following MGO treatment. However, pretreatment with LB attenuated the MGO-induced cytotoxicity (Figure 1A), apoptotic cell death (Figure 1B,C), and oxidative stress/ROS production (Figure 1D) of LLC-PK1 cells in a dose-dependent manner as LB alone did not show any toxicity. AG, a positive control, is known to inhibit the AGE formation. The mechanism involved trapping of intermediates at the initial glycation sages. Hepatotoxicity and drug resistance made its clinical use limited. Cell cytotoxicity assay indicates that with only LB pretreatment to cells, no toxicity was found. Although the LB prevents the MGO induced toxicity, however, the effect was less than the positive control drug. Pretreatment effect of LB on MGO induced-apoptosis indicates that reduced apoptotic cell death was noticed in early and late apoptosis. However, in early apoptosis, the effect was noticed in a dose-dependent manner and less than the positive control. While in late apoptosis, pretreatment with LB at the dose of 10 µg/mL was found to be more significant. However, the pretreatment effect of LB on apoptosis is less than the positive control (AG). In oxidative stress assay, pretreatment effect of LB in MGO induced oxidative stress was found to be significant at the dose of 20 µg/mL but less than the positive control.

### 3.2. LB Pretreatment Upregulated Expression of Glo1 and Nrf2 in MGO-Treated LLC-PK1 Cells

**Figure 2:**
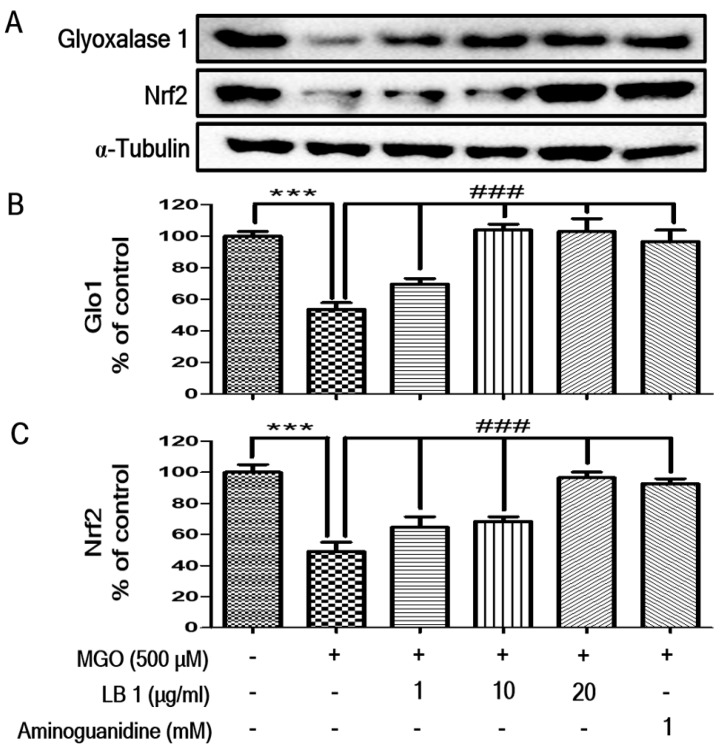
The effect of LB on Glo1 and Nrf2 protein expression. (**A**) Images showing the expression of Glo1 and Nrf2 proteins as measured by western blot analysis; and the relative band intensities of (**B**) Glo1 and (**C**) Nrf2. The bar values represent the mean ± SEM (## *p* < 0.01 and ### *p* < 0.001 vs. MGO treatment, and ** *p* < 0.01 vs. the control).

Western blotting revealed that the levels of Glo1 and Nrf2 decreased in LLC-PK1 cells following MGO treatment. However, pretreatment with LB upregulated the levels of Glo1 and Nrf2 in MGO-treated LLC-PK1 cells in a dose-dependent manner, suggesting that LB upregulated the expression of Glo1 by increasing the levels of Nrf2 in LLC-PK1 cells (Figure 2). Interestingly, pretreatment effect of LB on Glo1 and Nrf2 upregulation is more than the positive control.

### 3.3. Blood Glucose Tolerance

**Figure 3:**
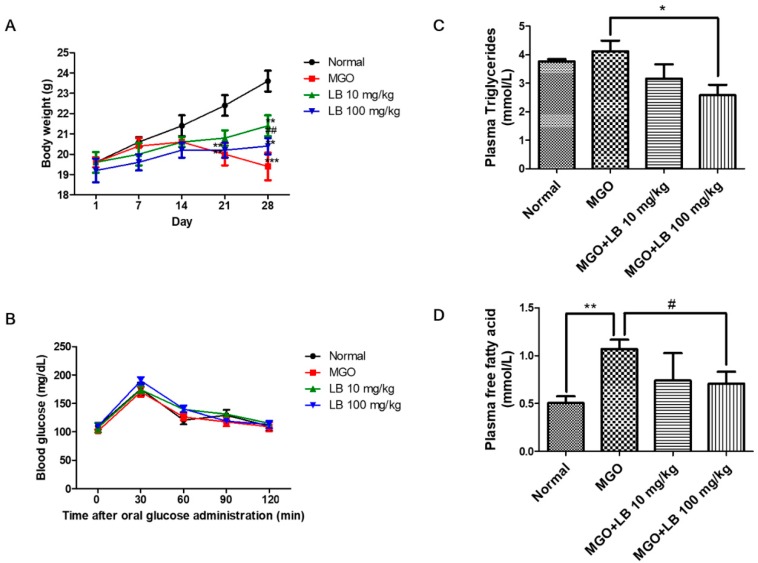
The effect of LB on MGO-induced increase of the plasma lipid profile. (**A**) BW of the experimental mice. (**B**) The results of OGTT (0−120 min) performed on the MGO-treated mice. (**C**) The plasma triglyceride levels of the MGO-treated mice. (**D**) The plasma levels of free fatty acids in the MGO-treated mice. The values represent the mean ± SEM (## *p* < 0.01 and ### *p* < 0.001 vs. MGO treatment, and ** *p* < 0.01 vs. the control).

Bodyweight (BW) of MGO-treated mice was significantly reduced, and LB pretreatment increased BW at the dose of 10 mg/kg of LB (Figure 3A). However, BW in the group treated with 100 mg/kg of LB did not show any significant difference. Fasting serum glucose levels did not change in any of the groups of mice (Figure 3B). These results suggested that short-term oral administration of MGO does not induce hyperglycemia in mice. 

### 3.4. LB Pretreatment Decreased the Levels of Plasma Lipids in MGO-Treated Mice

The levels of free fatty acids and plasma triglycerides significantly increased in MGO-treated mice. However, LB pretreatment decreased the levels of plasma triglycerides (Figure 3C) and free fatty acids (Figure 3D) in MGO-treated mice in a dose-dependent manner. Interestingly, LB pretreatment at a dose of 100 mg/kg was found to be most significant. 

### 3.5. LB Pretreatment Reduced MGO in the Kidneys of MGO-Treated Mice

**Figure 4:**
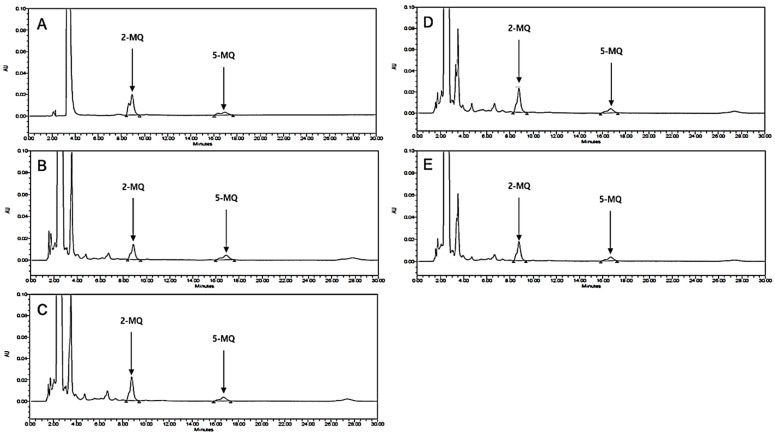
HPLC chromatograms of MGO in the kidneys of MGO-treated mice. (**A**) Mixture of the standard compounds. (**B**) Normal mice. (**C**) Mice treated with 300 mg/kg MGO. (**D**) Mice treated with MGO + LB (10 mg/kg). (**E**) Mice treated with MGO + LB (100 mg/kg).

MGO in the kidneys of the MGO-treated mice was quantified by HPLC-diode array detection (HPLC-DAD). As depicted in Figure 4, the level of MGO in the kidneys of MGO-treated mice was significantly elevated in comparison to that of the normal group mice. This elevated MGO level was significantly reduced following treatment with 100 mg/kg of LB (Table 1). These results indicated that LB pretreatment can decrease intracellular levels of MGO and protect against MGO-induced glucotoxicity. 

### 3.6. LB Pretreatment Decreased the Amount of AGEs in the Kidneys

**Figure 5:**
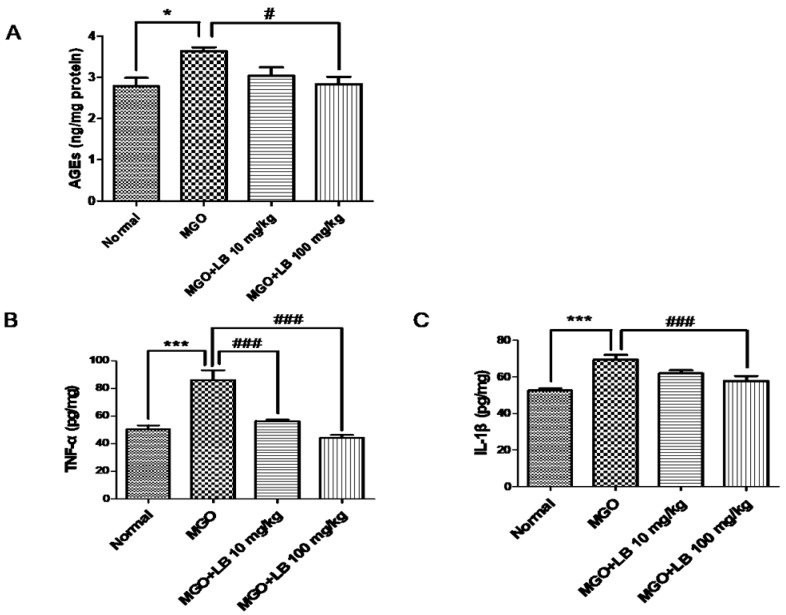
The effect of LB on MGO-induced elevation in the levels of AGEs and proinflammatory cytokines in the kidneys of MGO-treated mice. (**A**) Total amount of AGEs. (**B**) Levels of TNF-α. (**C**) Levels of IL-1β. The levels of AGEs and cytokines were determined by ELISA (ng/mg of protein). The bar values represent the mean ± SEM (# *p* < 0.05, ### *p* < 0.001 vs. MGO treatment, and * *p* < 0.05, *** *p* < 0.001 vs. the control, *n* = 6).

AGEs in the kidneys were quantified by an ELISA kit. The amount of AGEs in the kidneys was higher in the MGO-treated mice than in the normal mice (Figure 5A). However, treatment with 100 mg/kg LB significantly reduced the levels of AGEs in the kidneys. 

### 3.7. LB Pretreatment Attenuated the Levels of Inflammatory Cytokines in the MGO-Treated Mice

The upregulation of AGEs and RAGE, in turn, upregulated the levels of proinflammatory cytokines, including IL-1β and TNF-α, which play important roles in cell and tissue injury. Pretreatment with LB attenuated MGO-induced upregulation of proinflammatory cytokines in a dose-dependent manner (Figure 5). The result was significant when the dose of LB was 100 mg/kg. 

### 3.8. LB Pretreatment Upregulated Glo1 and Nrf2 in the Kidneys of MGO-Treated Mice

**Figure 6:**
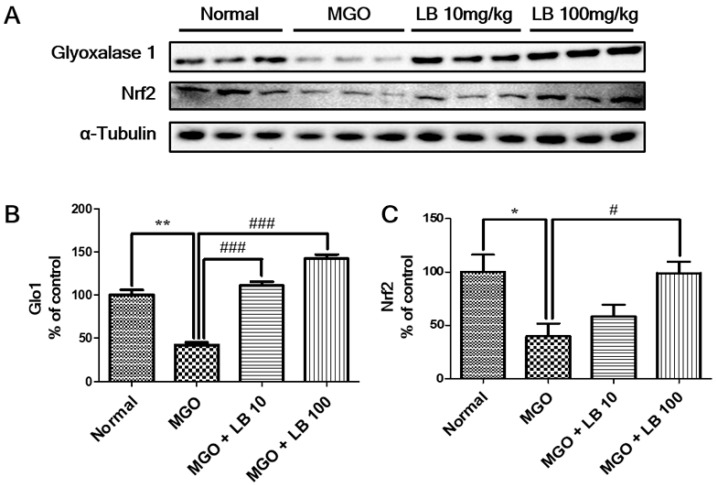
The effect of LB on the expression of Glo1 and Nrf2 proteins in the kidneys of the MGO-treated mice. (**A**) Expression of Glo1 and Nrf2 as measured by western blot analysis. (**B**,**C**) The relative band intensities of Glo1 and Nrf2. The bar values represent the mean ± SEM (# *p* < 0.05 vs. MGO treatment, and * *p* < 0.05, ** *p* < 0.01 vs. the control, *n* = 6).

Expression of Nrf2 and Glo1 was measured to determine whether treatment with LB was able to suppress MGO-induced glucotoxicity. Treatment with MGO significantly decreased renal expression of Nrf2 and Glo1 proteins (Figure 6). Pretreatment with LB restored expression of Nrf2 and Glo1 in the kidneys of MGO-treated mice. 

### 3.9. Pretreatment with LB Protected MGO-Treated Mice Against Renal Damage

**Figure 7:**
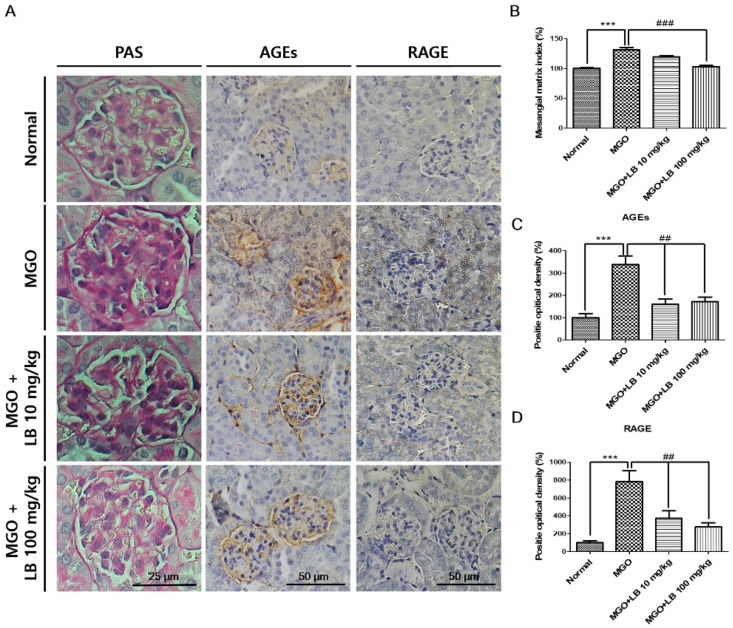
Immunohistochemical (IHC) and PAS staining of kidney tissues from MGO-treated mice. (**A**) Photomicrographs depicting PAS and IHC staining of kidney tissue sections of MGO-treated mice. (**B**) Quantification of PAS staining. (**C**,**D**) Quantification of IHC of AGEs and RAGE. The IHC- and PAS-stained histological sections are presented at magnifications of 200× and 400×, respectively. The bar values represent the mean ± SEM (# *p* < 0.05 vs. MGO treatment, and * *p* < 0.05, ** *p* < 0.01 vs. the control, *n* = 6).

In order to investigate whether LB has protective effects against renal damage, kidney tissues were subjected to periodic-acid Schiff (PAS) staining. The extent of fibrotic collagen in PAS-stained tissues was significantly higher in the MGO-treated mice than in the normal mice (Figure 7). However, staining intensity decreased in glomerular tissues of the LB-treated mice in a dose-dependent manner. These results indicate the potential protective effect of LB against renal damage in DN.

Immunohistochemical analysis revealed that treatment with MGO significantly increased renal levels of AGEs in comparison to those of normal mice, and that administration of LB markedly reversed this change (Figure 7). The increased intensity in staining corresponds with an increase in RAGE expression that is potentially increased in MGO-treated mice (Figure 7). It was further observed that expression of RAGE was limited or suppressed in the group that had been treated with LB following treatment with MGO. These results indicated that treatment with MGO induced the formation and accumulation of AGEs as well as RAGE, all of which were dramatically reduced with LB treatment.

### 3.10. HPLC Analysis of LB Extract 

**Figure 8:**
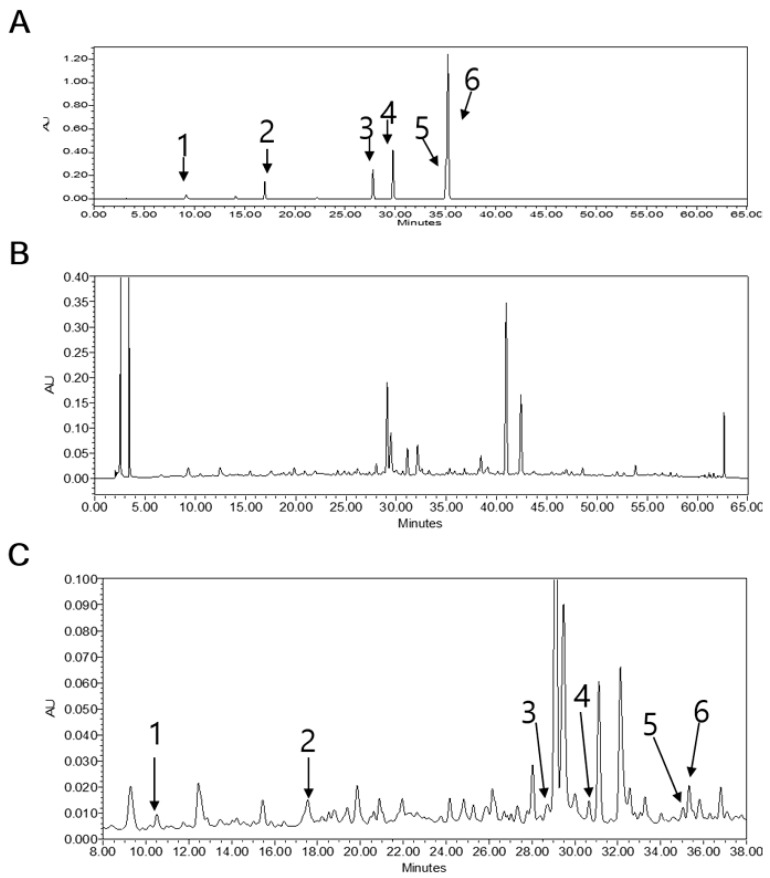
HPLC chromatogram. (**A**) Chromatogram of standard compounds mixtures: (**1**) catechin (Rt, 10.18 min), (**2**) rutin (Rt, 17.45), (**3**) daidzein (Rt, 28.83), (**4**) luteolin (Rt, 30.7), (**5**) naringenin (Rt, 35.28), (**6**) genistein (Rt, 35.32). (**B**) HPLC chromatogram of LB extract. (**C**) Close view of the chromatogram of LB extract.

HPLC-UV analysis revealed the major constituent and investigated peaks of LB extract were assigned as (1) catechin (Rt, 10.18 min), (2) rutin (Rt, 17.45), (3) daidzein (Rt, 28.83), (4) luteolin (Rt, 30.7), (5) naringenin (Rt, 35.28), and (6) genistein (Rt, 35.32) through comparing their retention time with the standard compounds in the chromatogram. Quantitative analysis indicated that LB consisted of 23.51 ± 0.17 µg/10 mg catechin, 43.69 ± 0.02 µg/10 mg rutin, 26.43 ± 0.06 µg/10 mg daidzein, 47.79 ± 0.06 µg/10 mg luteolin, 1.19 ± 0.02 µg/10 mg naringenin, and 11.54 ± 0.09 µg/10 mg genistein of powdered LB extract (Figure 8). 

## 4. Discussion

The different species belonging to the genus *Lespedeza* have anti-melanogenic, antidiabetic, and antioxidative properties [23,24]. Among the different plants belonging to the genus *Lespedeza*, *L. bicolor* (LB) has the highest number of active components that are effective against inflammation and oxidative stress [14]. LB is therefore consumed to cure diuresis, azotemia, and nephritis [14,23,24,25]. We recently reported that the stalks of *Lespedeza* sp. are a rich source of diverse bioactive phytochemicals that have a potential role against diabetic complications, such as endothelial dysfunction arising from MGO-induced glucotoxicity [13]. Although MGO plays a potential role in the development of DN, the therapeutic potential of LB against DN has yet to be explored. Therefore, the aim of this study was to explore and understand the therapeutic potential of LB in delaying the onset of DN.

MGO accumulation increases ROS generation, which leads to renal cell damage and apoptosis. Apoptotic death of renal cells is associated with different complications in DN. Therefore, protecting renal cells from aberrant apoptosis can offer a novel therapeutic strategy for treating DN [26]. MTT and apoptosis assays performed in this study confirmed that pretreatment with LB inhibits MGO-induced cytotoxicity and apoptosis, respectively (Figure 1). Based on these data, LB may protect renal cells against MGO-induced apoptosis. 

Nrf2 is an important transcription factor that regulates Glo1, a cellular enzyme involved in MGO detoxification [27]. Nrf2 activation upregulates Glo1 expression and protects renal cells against dicarbonyl stress [28]. In addition, Glo1 activity decreases in MGO-treated mesangial cells [29]. Therefore, targeting Nrf2 and Glo1 could be a new therapeutic avenue against MGO-associated cell damage. Furthermore, Yan et al. reported that MGO activates ROS, which triggers AGE-RAGE interactions that cause downstream cellular abnormalities to further hasten ROS production [30]. Additionally, during oxidative stress, ROS production increases that in turn increases the MGO level as well as AGEs formation that lead towards ROS-mediated cellular injury and death [31,32]. All this together causes inactivation of Nrf2 and downregulation of Glo1 [28]. 

Our in vitro experiments were consistent with these studies, demonstrating that pretreatment with LB inhibited intracellular production of ROS and upregulated Glo1 and Nrf2 expression in MGO-treated LLC-PK1 cells (Figure 2) suggesting the underlying signaling pathway to prevent DN.

Several studies have demonstrated that oral administration of MGO significantly increases plasma levels of blood glucose and free fatty acids in mice [33,34]. The accumulation of fatty acids and triglycerides plays a central role in the development and progression of diabetes [35]. Therefore, reducing plasma levels of blood glucose, free fatty acids, and triglycerides could offer another potential strategy for treating diabetes and its associated complications. The results of this study show that pretreatment with LB regulates blood glucose levels and the plasma lipid profile in MGO-treated mice (Figure 3), demonstrating the therapeutic potential of LB in treating diabetes and its associated complications. 

Previous reports suggest that administration of MGO markedly increases intracellular levels of MGO and enhances the formation of MGO-derived AGEs while decreasing plasma levels of Glo1 and Nrf2 in different animal tissues [36,37]. Based on HPLC analysis, we found that intracellular levels of MGO after oral administration were particularly high in the kidneys (Figure 4). The high levels of MGO triggered signaling pathways involved in DN progression. However, treatment with LB effectively reduced the levels of MGO and exhibited an antidiabetic effect that could ameliorate DM pathogenesis and its associated complications. 

MGO and AGEs trigger the inflammatory response upregulating the production of proinflammatory cytokines. Such inflammatory cascades provide clues to the onset of diabetes-related complications [38,39]. Therefore, reducing inflammatory cytokines can potentially ameliorate DM and its associated complications. As expected, treatment with LB reduced the level of AGEs and downregulated the expression of proinflammatory cytokines TNF-α and IL-1β (Figure 5). The treatment also promoted the expression of Nrf2 and Glo1 proteins by reducing MGO levels (Figure 6). Thus, LB treatment was protective against MGO-induced glucotoxicity and kidney damage in diabetic models in vivo. 

**Figure 9:**
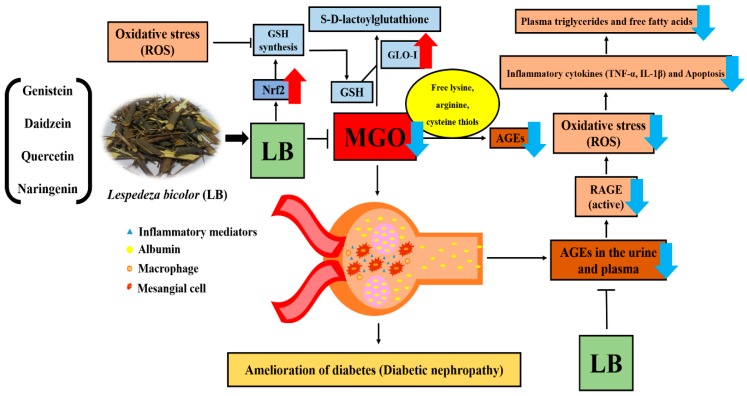
Mechanism of LB action on DM and its related complications. Treatment with LB significantly reduced oxidative stress, levels of plasma triglycerides and free fatty acids, apoptosis, inflammation (measured by the levels of IL-1β and TNF-α), the formation of AGEs, AGE-RAGE interactions, and renal damage. Pretreatment with LB also increased the levels of Glo1 and Nrf2 by suppressing the MGO levels. LB, therefore, prevented the occurrence of DM and its related complications, such as DN.

RAGE is a well-known AGE receptor, which comprises multiple-ligand receptors belonging to the immunoglobulin superfamily. Interactions between AGEs and RAGE initiate molecular signaling pathways, such as the MAPK signaling pathway. They also activate the transcription factor nuclear factor-κB that triggers the expression of proinflammatory cytokines, including TNF-α and IL-1β [40,41]. Moreover, AGE-RAGE binding causes albuminuria and mesangial expansion that ultimately results in glomerulosclerosis [4]. Our results support that pretreatment with LB reduces the apoptotic cell death, ROS production, lipid plasma level, inflammatory cytokines (IL-1β, TNF-α), AGE, and RAGE accumulation. In contrast, pretreatment with LB upregulates GLO1 and Nrf2 level. Collectively, these support that pretreatment with LB could be an effective therapy in prevention of DN (Figure 9). Among the species of plants belonging to the genus *Lespedeza*, *L. bicolor* significantly prevents the formation of AGEs and is also a rich source of flavonoids as revealed by HPLC-Q-TOF-MS/MS analysis [13]. Further standardization of the extract composition through HPLC analysis using our recently published experimental condition indicate that the major components of LB are catechin, rutin, daidzein, luteolin, naringenin, genistein, and naringin. Genistein, quercetin, and naringin were considered to inhibit the formation of AGEs, lipid profile, and ROS [13,42,43]. LB belongs to the legume family, therefore, isoflavone is high in LB like soybean, and also LB contains phenolic compounds including many different type flavonoid derivatives. These factors support that preventive effect of LB against diabetes might be due to the presence of these active constituents [13]. However, the effectiveness of these components, as shown by LB against MGO related signaling pathway in diabetic nephropathy, need to be explored.

The results of this study demonstrated that MGO increased ROS production, the formation of AGEs and RAGE, apoptotic cell death, plasma levels of glucose, and fatty acids, and expression of pro-inflammatory cytokines such as TNF-α and IL-1β. In contrast, MGO decreased the expression of Glo1 and Nrf2, which are common, interlinked causative factors responsible for initiating different pathological conditions associated with DN and severe renal damage [4,5,7,13,26]. LB treatment can counter these effects by significantly reducing oxidative stress, apoptosis, inflammation, and renal damage, as well as increasing the levels of Glo1 and Nrf2 by regulating the levels of MGO, which prevented the occurrence of DM and its related complications, as depicted in Figure 9.

Taken together, the results of the experiments performed herein indicate that *L. bicolor* could be effective for treating diabetic nephropathy arising from MGO-induced glucotoxicity.

## 5. Conclusions

Our study indicates that *L. bicolor* recovers MGO-induced metabolic dysfunction and glucotoxicity in diabetic nephropathy by reducing oxidative stress, inflammation, and renal damage by upregulating Glo1 and Nrf2. However, since *L. bicolor* extract is composed of several components that could act synergistically to prevent diabetic nephropathy, additional work is needed to identify the potentially complex multi-target mechanisms.

## Figures and Tables

**Table 1:** MGO levels in MGO-treated mouse kidneys.

Sample	Concentration (nmol/mg Protein)
Normal	0.972 ± 0.148
MGO (300 mg/kg)	1.704 ± 0.053 **
MGO (300 mg/kg) + LB (10 mg/kg)	1.680 ± 0.063
MGO (300 mg/kg) + LB (100 mg/kg)	1.152 ± 0.050 ^##^

The values represent the mean ± SEM (^##^ *p* < 0.01 vs. MGO treatment, and ** *p* < 0.01 vs. the control).

## Abbreviations

LB	*Lespedeza bicolor*
MGO	methylglyoxal
ROS	reactive oxygen species
AGEs	advanced glycation end-products
RAGE	receptor for advanced glycation end products
Glo1	glyoxalase 1
Nrf2	nuclear factor erythroid 2-related factor 2
DM	diabetes mellitus
DN	diabetic nephropathy
ARE	antioxidant responsive element
o-PD	o-phenylenediamine
BSA	bovine serum albumin
MTT	3-(4,5-dimethylthiazol-2-yl)-2,5-diphenyltetrazolium bromide
DMSO	dimethyl sulfoxide
FBS	fetal bovine serum
DMEM	Dulbecco’s modified Eagle’s medium
2-MQ	2-methylquinoxaline
5-MQ	5-methylquinoxaline
BW	body weight
HPLC	high-performance liquid chromatography
FACS	fluorescence-activated cell sorting
